# Early Mobilization in the Treatment of War Wounds

**Published:** 1919

**Authors:** 


**Early Mobilization in the Treatment of War Wounds.**
By Dr. P. Kouindjy. *Paris Médical,* October 5, 1918.

It sems that, after 4 years of war, the utility of early mobilization in the treatment of war wounds is no longer open for discussion. An approximate comparison between the numbers of cases of ankylosis and impotence due to muscular atrophia in 1915 and 1918 shows a real improvement in our methods of treatment. It must, however, be admitted that this improvement is not so general as it might be, and that troublesome cases still occur.

The author gives in detail a case of ankylosis and stiffness of the finger and wrist joints, together with fibrous ankylosis of the shoulder. This case, resulting from a panaris on the fourth finger, complicated by a phlegmon on three fingers, he declares is explained by the prolonged fixation of the patient's arm. During the first five months of treatment, the surgeons were so taken up by the wound itself that no notice was taken of the glenohumeral joint. The author previously drew attention to ankylosis of the shoulder caused by a fracture of the radius, and to that of the instep resulting from a seton wound either in the thigh or in the gluteal region.

In order to explain the pathologic phenomena due to prolonged immobilizition, he quoted the work on trophic troubles caused by excessive fixation of the joints, published in 1841 by Teissier. He maintains now that, in the above described case, the shoulder joint underwent the two first ankylosis processes described by Teissier : (1) Phasis of sero-sanguinous secretion; (2) Fibrous transformation of this matter and formation of false membranes.

The chief objections brought forward by ankylophiles against early mobilization of war traumatisms are that, in their opinion, it delays the consolidation of fractures, prevents cicatrization and increases suppuration. These objections cannot hold after careful study. Injured joints must not be mobilized before complete consolidation of the fracture, but joints further removed should be mobilized from the beginning of the treatment, and the same should be applied, after consolidation of the fracture, to the injured joint and to those in its neighborhood.

The author then gives a case of musculo-articular impotence due to an open fracture of the upper third of the humerus, with wide, deep wound, smashed diaphysis and atrophia of the muscles of the arm, in which the normal functions of the limb were completely restored after 2 1/2 months of treatment, thanks to early mobilization of the joints.

A comparison between the two cases here recorded clearly shows the desirability of early and systematic mobilization in the treatment of war wounds. In the first instance, the patient wounded on March 9th, 1917, is still under treatment in October, although the original injury was but a panaris on the 4th finger; in the second, the patient suffering from a complicated fracture of the humerus was fit for active service on September 25th.

Conclusions: (1) All war wounds should be submitted to timely mobilization.

(2) Functional recovery of a joint should never be left to nature.

(3) Mobilization by hand should not be confused with mechanotherapy, which is often harmful. Progressive and methodical movement of injured joint can only be obtained by hand.

